# The Effect of Extrafascial Hysterectomy After Completion of External Beam Radiotherapy for Treatment of Locally Advanced Stages (IIB-III) of Cervical Cancer

**DOI:** 10.5812/ircmj.10758

**Published:** 2013-12-05

**Authors:** Zahra Sarraf, Bahareh Hamedi, Soodabeh Hooshmand, Ahmad Mosalaie, Minoo Robati, Mozhdeh Momtahan, Pouya Farhadi

**Affiliations:** 1 Gynecologic Oncology Ward, Department of Obstetrics and Gynecology, Shiraz University of Medical Sciences, Shiraz, IR Iran; 2 Department of Radiotherapy and Oncology, Shiraz University of Medical Sciences, Shiraz, IR Iran; 3 Trauma Research Center, Shiraz University of Medical Sciences, Shiraz, IR Iran

**Keywords:** Uterine Cervical Neoplasms, Lymphatic Irradiation, Hysterectomy

## Abstract

**Background:**

Worldwide, cervical cancer is one of the most challenging gynecologic cancers in treatment.

**Objectives:**

This study was designed with the aim of comparing patients treated with External Beam Radiotherapy (EBRT) and Interactivity Brachytherapy (ICBT) with EBRT and extrafascial hysterectomy in locally advanced stages of cervical cancer (IIB-III).

**Patients and Methods:**

The present study was designed as a case-control which was performed on the patients with cervical cancer in locally advanced stages (IIB-III) admitted to Namazi and Faghihi hospitals (university hospitals in Shiraz) between 2008-2011. 51 patients were included in two distinct groups: 25 patients were treated with EBRT and Interactivity Brachytherapy (group A). 26 patients were treated with EBRT and extrafascial hysterectomy group B.

**Results:**

In group A, the number of patients with FIGO stage IIb and III were 16 and 9, respectively, and 17 and 9 in group B. The median duration of follow-up was 24 months. There were no significant differences between two groups in metastasis and recurrence rate (P > 0.05). 5-years overall survival rate was 54.8% [95% CI: 39-70.9] in group A and in group B was 50.9% [95% CI: 41.5-60] and The LOG-rank test which controls the effect of treatment modalities on overall survival rate, did not show any significant difference between two groups (P = 0.407).

**Conclusion:**

The results of our study showed that the trend of treatment using EBRT along with intracavity brachytherapy may have the same outcome as the method of using EBRT and extrafascial hysterectomy. Overall, it seems that external beam radiation followed by extrafascial hysterectomy could be a proper substitute for brachytherapy.

## 1. Background

Worldwide, cervical cancer attracts enormous importance as a public health issue. In terms of frequency, cervical cancer represents the second place in women all around the world causing approximately 237500 deaths annually, and generally it is the seventh cancer in the list of most frequent cancers worldwide. In the year 2010, approximately 12200 newly diagnosed cervical neoplasms were recorded in the USA of which, 4210 deaths were resulted. Unfortunately, in nations that do not have efficient cervical cancer screening Programs this malignancy persists to be in the second place in terms of frequency (17.8 per 100,000 women) (1).

Cervical transformation zone is the anatomic site which nests the malignancies of cervix. The surrounding tissues, such as the vagina, side walls of the pelvis or nearby lymph nodes are the anatomic sites where this cancer can nest by metastasis (2). Speaking of local neoplasms and metastatic tumors of cervical origin, there are two important factors (size and location of the tumor) which should be taken into consideration in determining the precise treatment of option. Regarding the treatment of locally advanced cervical cancers, Surgery, radiotherapy or sometimes both modalities are considered to be the treatments (3). In the past, standard treatment modalities were radiotherapy or radical hysterectomy with 5 year survival rates of 80 to 90% (4). Respecting the radiotherapy of cervical cancer, the current paradigm implies that intracavitary brachytherapy plays an extremely important role in radical treatment of cervical cancer. On the other hand, for many years many organizations routinely used adjuvant extrafacial hysterectomy for bulky exophytic or "barrel" shaped tumors. This process has been gradually abandoned as a randomized research reveals no advantage on survival of adjuvant hysterectomy (5). A good possibility of treatment was with pelvic radiotherapy, but the highest radiation dose that was given to patients was restricted by normal tissue tolerance, particularly of the small bowel, rectum and bladder (3).

In 1999, it was an alert by The National Cancer Institute, declaring that concomitant chemoradiation should be used in females with cervical cancer instead of radiotherapy alone. This statement was supported by results of large randomized trials and a meta-analysis .Thus, for treating locally advanced cancers, the new standard of care has changed to concomitant chemoradiotherapy (6). Today, women suffering from this cancer, benefit from surgery (removing the womb) as well as combined chemotherapy and radiotherapy (7, 8). A well-known prognostic aspect in cervical cancer is the pathological complete response obtained with preoperative treatment (8, 9). It is at least intriguing that a retrospective study of the pathological response rates in patients undergoing adjuvant surgery after primary external beam radiation with and without brachytherapy demonstrates no significantly differences (10).

Overall, in studies using external beam radiation (EBRT) at doses between 37.4 to 52Gy, in common fractions of 1.8 or 2Gy daily, plus brachytherapy, the average of pathological response rate observed was 50%, whereas in those using EBRT-CT at similar doses with either weekly cisplatin or the combination of cisplatin and 5-fluorouracil plus brachytherapy, the corresponding average is 51.1% (11-17). Interestingly, in four trials using EBRT-CT but no brachytherapy, the pathological response rate is essentially the same, a mean of 51.6% (45%, 45.2%, 46.6%, 54.2 and 67%) (18-21). These data are quite provocative and recommend that for these stages, brachytherapy could be unimportant, however, it must be stressed that such comparison is based on highly heterogeneous studies and as such data is only generating hypothesis (22). 

## 2. Objectives

Regarding that data is controversial in management of locally advanced cervical cancers; this study was designed with the aim of comparing patients treated with External Beam Radiotherapy (EBRT) followed by Intracavity Brachytherapy (ICBT) with EBRT followed by extrafascial hysterectomy in locally advanced stages of cervical cancer (IIB-III).

## 3. Patients and Methods

The present study was designed as a case-control study which was performed on female patients with cervical cancer in locally advanced stages (IIB-III). These patients were admitted to Namazi and Faghihi hospitals (university hospitals in Shiraz, Iran) from January 2008 to January 2011. The study examined and compared patients treated with EBRT followed by ICBT with EBRT followed by extrafascial hysterectomy. The study was approved by the Shiraz University of Medical Sciences Ethics Committee (Ethical number: 12984-E, at October 12, 2007). All patients had previously untreated and histologically confirmed cervical cancer. Inclusion criteria were: FIGO stage IIB- III (locally advanced) cervical cancer, age < 75 years, normal liver function, normal kidney function, no prior cancer.

Patients with the following characteristics were excluded from the study:

A. Patients treated with other chemoradiotherapy regimens.

B. Patients with other malignancies.

Informed consent was taken from all patients in the first visit. Complication of radiotherapy, surgery and risk of morbidity and mortality were mentioned in the consent and discussed with all of the patients.

### 3.1. Selection of Participants

The individuals participating in the study were divided into two matched groups (group A and B). The regimen for radiation therapy in group A was EBRT of a total dose of 50Gy delivered at 2Gy per day, 5 days a week for 5 weeks and for these patients ICBT was performed using a dose of 10Gy per week administered for a total dose of 30Gy. In group B, EBRT was performed in the same way as group A and patients underwent extrafascial hysterectomy four to six week after radiotherapy. There were some reasons for substitution of ICBT by hysterectomy in group B including: functioning defects of the megavoltage machine used for radiotherapy, patients’ refusal, cervical OS stenosis and vaginal stenosis in some cases.

### 3.2. Data Collection

Baseline characteristics of the patients including parity, gravid, number of live births or abortions, FIGO stage and histologic type of cervical cancer were collected using a data gathering form. Mortality rate was considered as the primary outcome. Post-treatment morbidity, Metastasis occurrence, residual tumor and local recurrence rate as the secondary outcomes were also considered. The local recurrence rate was estimated according to the FIGO stage.

### 3.3. Statistics

All statistical analyses were performed with the Statistical Package for Social Sciences version 17.0 (SPSS Inc., Chicago, IL, USA). The baseline characteristics of both treatment groups were compared using X2 tests or Fisher’s exact test for proportions and unpaired t-tests for means. The overall survival was estimated by Kaplan-Meier method and survival curves were compared using the Log-rank test. The 95% confidence intervals for the means of data were calculated, and the significance of differences was assessed using the unpaired Student’s t-test.

## 4. Results

### 4.1. Baseline Characteristics

Sixty-two patients fulfilled the inclusion criteria, out of which 11 patients were excluded from the study (6 from group A and 5 from group B) because of unsuccessful follow ups. Finally, 51 patients were included in two distinct groups: 25 patients were treated with External Beam Radiotherapy (EBRT) and Intracavity Brachytherapy (group A). 26 patients were treated with EBRT and extrafascial hysterectomy (group B). Baseline characteristics of patients including age, parity, gravid, number of live birth, FIGO stage and histopathologic type of cervical cancer were similar in two groups as it is represented (Table 1). 

**Table 1.** Baseline Characteristics of Patients

Variable	Group A, Mean (SD) or absolute number	Group B, Mean (SD) or absolute number	P Value
**Age**	56.4 ± 11.9	54.5 ± 9.9	0.531
**Gravid**	5.48 ± 2.2	4.8 ± 2.2	0.292
**Live birth**	4.38 ± 2.36	5.24 ± 2.24	0.192
**FIGO Stage**			0.918
IIb	16 (64%)	17 (65.4%)	
III	9 (36%)	9 (34.6%)	
**Histology**			0.977
SCC	24 (96%)	25(96.6%)	
Adenocarcinoma	1 (4%)	1 (3.8%)	

### 4.2. Outcomes

In group A, the number of patients with FIGO stage IIb and III were 16 and 9, respectively, and 17 and 9 in group B. 12 patients showed residual disease after extrafascial hysterectomy in group B. The median follow-up time in group A was 16.5 months (ranged 2.7-64 months), during which the disease recurred in 9 patients (36%), out of which eight patients died of recurrence. In group B, median follow-up time was 29 months (range, 2-83 months), during which the disease recurred in seven patients (26/9%) of which five died of recurrence. Regarding recurrence, data analysis showed no significant differences between two groups (Table 2). 

**Table 2.** Comparison of Number of Recurrence and Metastasis Between two Groups

	Group A, No. (%)	Group B, No. (%)	P value
**Recurrence**	9 (36%)	7 (27%)	0.485
**Metastasis**	4 (16%)	2 (7.7%)	0.413

**Figure 1.**
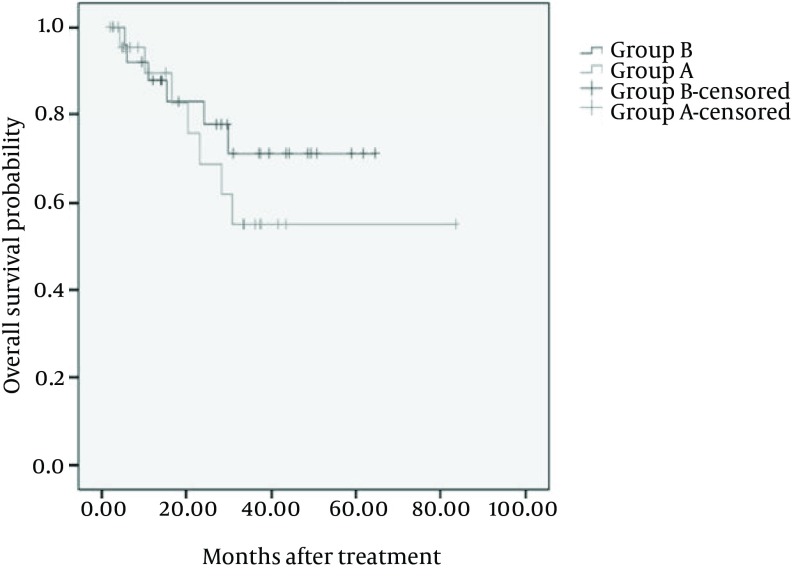
Kaplan-Meier Curves of 5-years Overall Survival in Both Groups

In group B, metastases were observed in 15/4% (4) of the patients while in 8% (2) of the patients in group A, metastasis occurred. Overall, there was no significant difference between two groups (Table 2). In group A, Post-treatment morbidities were observed in 5 cases including: hydronephrosis (2), DVT (2), vasicovaginal fistula (1) and there was one case with Post-treatment morbidity which was hydronephrosis in group B. Overall, the median duration of follow-up was 24 months. Kaplan-Meier estimated 5-years overall survival rate was 54.8% [95% CI: 39-70.9] in group A and 50.9% [95% CI: 41.5-60] in group B. 5-years overall survival curves, are shown in Figure 1. The LOG-rank test which controls the effect of treatment modalities on overall survival rate, did not show any significant difference between two groups (P = 0.407). 

Speaking of cervical cancer treatment methods, highly specific trends exist regarding the definite staging of the disease using FIGO staging system. Performing total hysterectomy or a local procedure such as conization is the recommended treatment for early stage cervical cancer (IA1); whereas, for IA2 patients, the recommendation is for a radical hysterectomy which removes parametrial tissue, upper vagina and pelvic lymph nodes. Because many women at this disease stage wish and deserve to preserve fertility, radical trachelectomy is becoming an option for these patients as well as for IB1 patients (23). Evidence for the prognostic significance of ICBT in cervical cancer is concluded mainly from retrospective studies. In a large investigation involving 1096 patients with FIGO stage III cervical cancer, 5-year disease-free survival rates were 27% with EBRT compared with 53% using EBRT followed by ICBT (P < 0.0001) (24).

Regarding the treatment choices for locally advanced cervical cancers, it should be mentioned that treatment of this stage has experienced no major changes for nearly 80 years during which exclusive radiation was considered the standard of care. However, over the last 20 years, a number of trials testing concurrent chemoradiation were performed in an attempt to improve treatment results (25, 26). It was not until 1999 that results of five randomized studies including nearly 2,000 patients were published, demonstrating that survival rate with concomitant chemotherapy (RT/CT) based on cisplatin was superior than that obtained with radiation alone (26). The results of these five trials led the National Cancer Institute of the US to issue an alert recommending the addition of cisplatin-based chemotherapy to patients who receive radiation as primary treatment. In this regard, another question exists which is around the role of hysterectomy after chemoradiotherapy in management of cervical cancer. Reviewing the literature reveals that early stage bulky tumors can be managed with either primary chemoradiation (external and brachytherapy) or radical hysterectomy and tailored adjuvant radiation or chemoradiation. Extensively commented in literature, both methods have supporters and detractors (27, 28).

On the contrary, evaluation of the role of radical hysterectomy after either external beam radiation or chemoradiation has been done only by a few numbers of studies. Such studies are not randomized trials. In the year 1993, in an investigation of 20 uterine cancer patients with bulky disease FIGO staged as I (50%), II 45% and 5% stage III, radical hysterectomy after definitive radiation was done because patients were at high risk for recurrence. Morbidity of radical hysterectomy after radiation was declared by the authors but they conclude that it might be effective in treating patients with 1) uterine sarcomas involving the cervix 2) large cervical tumors 3) small recurrent cervical tumors 4) patients unable to undergo brachytherapy for cervical cancer and 5) cervical cancer that responds poorly to radiation (29). In another larger study of 187 patients, evaluation of radiotherapy followed by radical surgery including systematic para-aortic lymphadenectomy was done. Overall survival at 3 years was 85%, 56%, and 40% in patients with negative nodes, positive pelvic nodes, and positive para-aortic nodes, respectively although there were surgical complications in 18% of patients (30). Reviewing a more recent study of locally advanced cervical cancer in 30 patients shows that adjuvant surgery may improve the outcome of patients with bulky residual tumor after chemoradiation, allowing a 5-year survival of 55.6% after curative intervention (31). At a median follow- up of 22 months, another study however, reported that the disease free individuals were only two out of a total number of 10 patients (32).

The results of another non-randomized comparison conducted by Cetina et al. (22), in which 80 patients, receiving EBRT-CT and surgery suggest that external beam chemoradiation using cisplatin followed by brachytherapy or a radical hysterectomy and tailored brachytherapy offers the same survival probability .These results along with the existing data in literature are very suggestive that brachytherapy may be dispensable in early stage cervical cancer patients as long as radical hysterectomy with pelvic lymphadenectomy is performed. The evidence showing that after definitive radiation between 11 and 20% of patients will be left with positive pelvic lymph nodes that remain untreated if are not removed by surgery, poses an additional potential advantage of surgical treatment (33).

There are other benefits with surgical management of patients with cervical cancer which will be reviewed briefly. Traditionally, maximizing the overall survival of patients has been the mainstay of oncologists’ concern. Quality of life (QOL) after cancer therapy is an important aspect of patient care; although, in time of recommending cancer treatment, it is often not the main consideration by the oncologists. However, when equivalent clinical outcomes exist for various competing treatment options, QOL considerations become particularly important (34). It is currently known that, cervical cancer survivors treated with radiotherapy had worse sexual functioning than those treated with radical hysterectomy and lymph node dissection (34). This could be mentioned as an area in which, radical hysterectomy gains superiority over radiotherapy regimens.

 It should be mentioned that delivery of treatment in the field of radiotherapy as a multidisciplinary specialty requires complex equipment and radiation sources. It is estimated that currently, in developing countries over 2100 megavoltage teletherapy machines are installed. This figure is significantly below the estimated current needs of almost 5000 machines. By the year 2015, about 10,000 machines will be needed as a conservative estimate points to. It should also be considered that besides the limitations in equipments, there is an enormous need for qualified professionals (including radiation oncologists, medical physicists, radiotherapy technicians, radiation protection officers, and maintenance engineers) which unfortunately worsens the picture. Just as examples, only 22 out of 56 countries in Africa were known to have megavoltage therapy and the population served by each megavoltage machine ranges from 0.6 million to 70 million per machine (35). The situation in the Asian Pacific countries was not much better, with significant deficiencies in the availability of all components of radiation therapy as it is in Latin America where not only there is a shortfall of equipment, but also a major restriction to patient service is an insufficient number of specialists (36, 37).

Considering the above mentioned issues, it would be obvious that locally advanced cervical cancer patients who live in countries with limited resources, may not have sufficient radiation therapy resources and other therapeutical options are needed for such patients (11). Our center (Faghihi hospital) as a large referral center in the southern part of Iran possesses only one megavoltage machine, performing brachytherapy for cervical cancer patients. Considering the fact that a functioning defect compromised the machine performance, our center failed to deliver radiotherapy to individuals requiring it for a period of time. Therefore, in this situation, EBRT followed by extrafascial hysterectomy as the surgical treatment of choice were used for patients diagnosed with locally advanced cervical cancer. Moreover, at the time which our machine was performing correctly, there were some patients who could not go on radiation therapy. The section on the method of our study has provided related information for this issue. 

Interestingly, due to results of our study, post treatment complications observed with extrafascial hysterectomy were of little importance comparing to those with brachytherapy. Overall, it seems that external beam radiation followed by extrafascial hysterectomy could be a proper substitute for brachytherapy in this situation, reducing the need for such treatment modality. The main limiting factors of our study include the small number of patients and its retrospective nature. Another potential source of bias was that patients with stage IVa were not included in the study as locally advanced cancer. Finally, it is recommended that further research projects, especially randomized clinical trials involving larger number of patients should investigate more factors such as patients’ quality of life and their sexual functioning status.

## 
